# αβγδ T cells play a vital role in fetal human skin development and immunity

**DOI:** 10.1084/jem.20201189

**Published:** 2021-02-09

**Authors:** René Reitermaier, Thomas Krausgruber, Nikolaus Fortelny, Tanya Ayub, Pablo Augusto Vieyra-Garcia, Philip Kienzl, Peter Wolf, Anke Scharrer, Christian Fiala, Marita Kölz, Manuela Hiess, Martin Vierhapper, Christopher Schuster, Andreas Spittler, Christof Worda, Wolfgang Weninger, Christoph Bock, Wolfgang Eppel, Adelheid Elbe-Bürger

**Affiliations:** 1 Department of Dermatology, Medical University of Vienna, Vienna, Austria; 2 Research Center for Molecular Medicine (CeMM) of the Austrian Academy of Sciences, Vienna, Austria; 3 Department of Dermatology, Medical University of Graz, Graz, Austria; 4 Department of Pathology, Medical University of Vienna, Vienna, Austria; 5 Gynmed Clinic, Vienna, Austria; 6 Department of Women’s and Children’s Health, Division of Obstetrics and Gynaecology, Karolinska Institute and Karolinska University Hospital, Stockholm, Sweden; 7 Department of Urology, Medical University of Vienna, Vienna, Austria; 8 Department of Surgery, Division of Plastic and Reconstructive Surgery, Medical University of Vienna, Vienna, Austria; 9 Core Facilities, Flow Cytometry, Medical University of Vienna, Vienna, Austria; 10 Department of Obstetrics & Gynecology, Medical University of Vienna, Vienna, Austria; 11 Department of Laboratory Medicine, Medical University of Vienna, Vienna, Austria

## Abstract

T cells in human skin play an important role in the immune defense against pathogens and tumors. T cells are present already in fetal skin, where little is known about their cellular phenotype and biological function. Using single-cell analyses, we identified a naive T cell population expressing αβ and γδ T cell receptors (TCRs) that was enriched in fetal skin and intestine but not detected in other fetal organs and peripheral blood. TCR sequencing data revealed that double-positive (DP) αβγδ T cells displayed little overlap of CDR3 sequences with single-positive αβ T cells. Gene signatures, cytokine profiles and in silico receptor–ligand interaction studies indicate their contribution to early skin development. DP αβγδ T cells were phosphoantigen responsive, suggesting their participation in the protection of the fetus against pathogens in intrauterine infections. Together, our analyses unveil a unique cutaneous T cell type within the native skin microenvironment and point to fundamental differences in the immune surveillance between fetal and adult human skin.

## Teaser

By characterizing the poorly defined T cell composition in developing human fetal skin, Reitermaier et al. identify a unique population of TCR αβγδ+ T cells. These cells may contribute to early skin development and immune defense against invading pathogens in utero.

## Graphical Abstract

**Figure:**
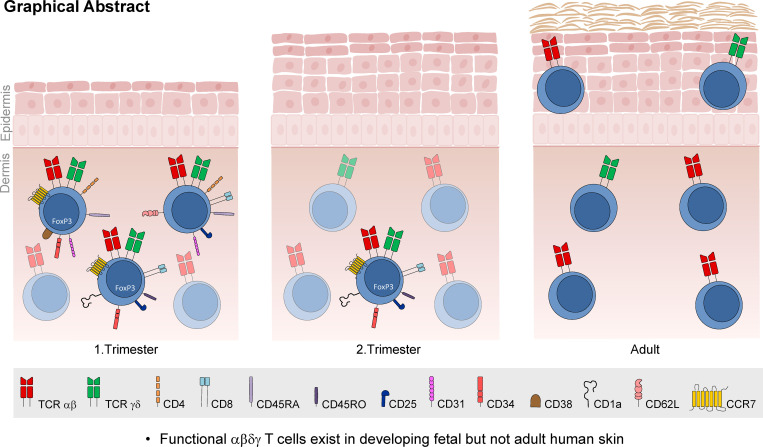

## Introduction

T cells are defined by the expression of heterodimeric TCRs composed of either α and β or γ and δ chains. The disparate T cell lineages respond to distinct antigens and act in concert not only to survey a wide range of antigens to enable protective immunity but also to modulate the function and behavior of other (immune) cells. The developing conceptus is, in principle, protected from pathogens by the uterine barrier and maternal-derived antibodies. Nonetheless, the sterile environment of the amniotic cavity may be breached, and it is therefore essential that organs such as the skin establish a well-functioning immune network to provide immune defense against invading pathogens. This is initiated together with the structural differentiation and maturation of skin and continues to proceed throughout human life. T cells, which are known to inhabit fetal skin (Di Nuzzo et al., 2009; Schuster et al., 2012; Sanchez Rodriguez et al., 2014), remain poorly characterized in terms of their development, lineage relationship, phenotype, and function.

## Results and discussion

**Figure 1.**
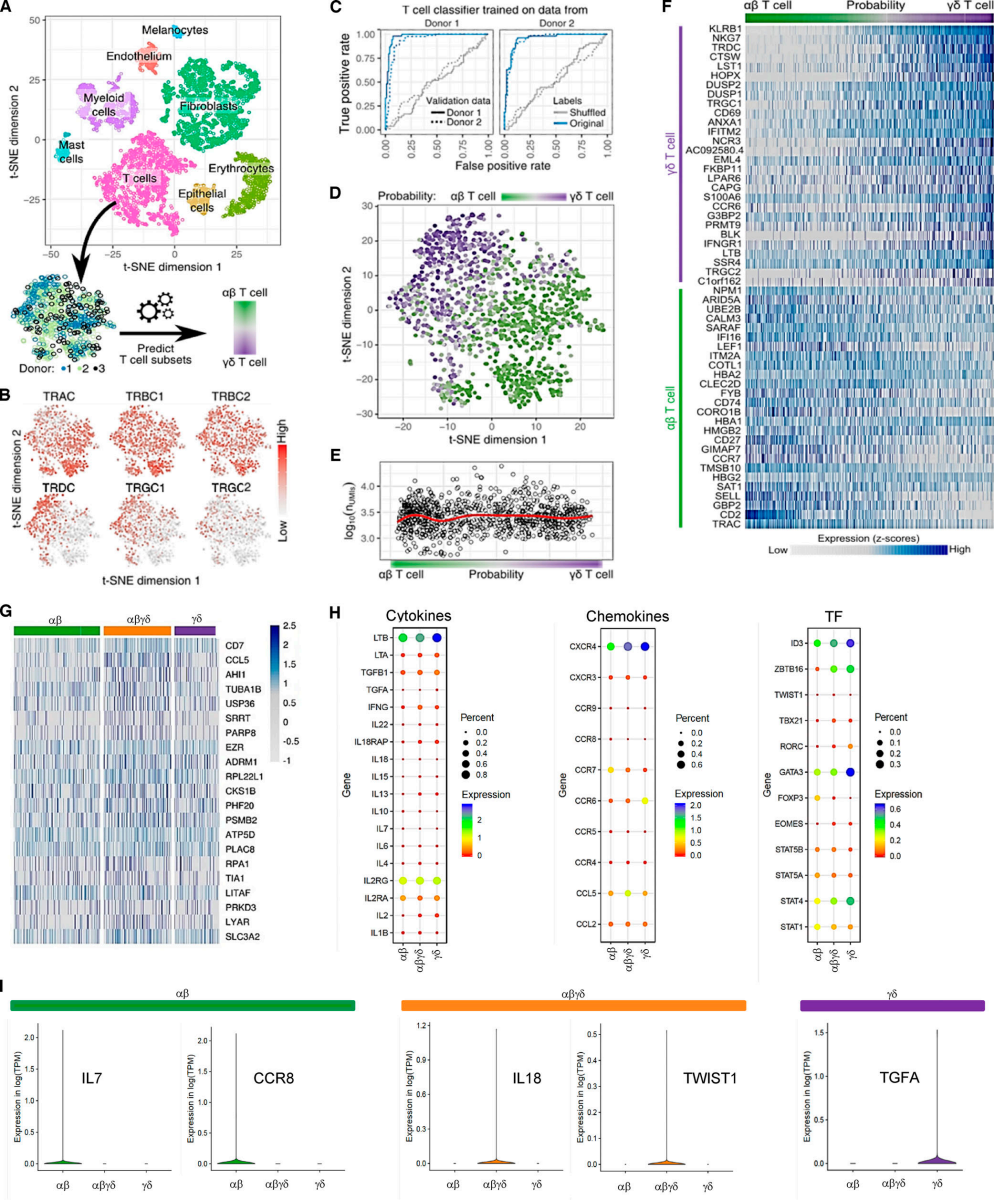
**Identification of an unconventional T cell population in fetal skin using single-cell analyses. (A)** Clusters in the t-SNE blot were assigned to all identified cell types. From the full dataset, T cell subsets were extracted throughout three donors (17–22 wk EGA). **(B)** Expression of TCR subunits across T cell subsets. **(C)** Prediction accuracy for αβ and γδ T cells from expression data are demonstrated as the ROC curve. Predictors of one donor were tested on the same donor using nested cross-validation and across donors. Prediction accuracy was also compared with data with shuffled labels. Probability scores of 0 and 1 indicate αβ and γδ T cells, respectively. **(D)** t-SNE clustering of T cell subsets from three donors, with color-coded probability of cells representing αβ or γδ T cells. **(E)** T cell probability compared with data quality, measured as the number of UMIs. Red line shows a local regression fit through data. **(F)** Marker genes in αβ and γδ T cells. **(G)** Heatmap showing highest gene expression levels in DP αβγδ fetal skin T cells in comparison to SP T cell subsets. **(H)** Dot plots of fetal skin T cell subsets displaying average gene expression (colors) and frequency (circle size) of selected cytokines, chemokines, and transcription factors (TF). **(I)** Exclusive expression of indicated genes in SP and DP T cell subsets. Means of the average expression levels are indicated by color.

We performed a comprehensive study to gain insight into the developing immune milieu of human skin and, in particular, to investigate the nature of prenatal cutaneous T cells. We transcriptionally profiled single cells from second trimester fetal skin (17–22 wk estimated gestational age [EGA]) and captured nonimmune and immune cells. Cell clustering using t-distributed stochastic neighbor embedding (t-SNE) enabled the clear distinction of major cell types including T cells (Fig. 1 A). Using unsupervised clustering of TCR-expressing cells, we discovered an intermediate cell population that coexpressed both αβ and γδ TCR constant chains, indicating the unexpected existence of double-positive (DP) αβγδ T cells (Fig. 1 B). To interrogate the relationship of these cells with single-positive (SP) T cells at the whole transcriptome level, we developed a machine learning–based classifier that classifies cells as either αβ or γδ T cells using the expression of all genes. Predictions from this classifier were highly accurate (Fig. 1 C) based on receiver operating characteristic (ROC) curves. The classifier identified a spectrum of T cells spanning the expected SP αβ and γδ T cell subsets as well as a previously unrecognized intermediate DP αβγδ T cell population (Fig. 1 D), independently of data quality measures such as the number of unique molecular identifiers (UMIs; Fig. 1 E). These intermediates coexpressed marker genes typical for SP αβ and γδ T cell subsets (Fig. 1 F), but also particular genes in higher abundance than observed in both SP T cell subsets (Fig. 1 G). The high cellular frequency of DP αβγδ T cells was robust and stage specific and was validated with orthogonal methods (Fig. 3). All three subsets displayed gene expression profiles typical for type 1 immune cells (lymphotoxin β, IFN-γ, and STAT4), regulatory T (T reg) cells (TGF-β, STAT5, and CCR4), and regulators of hematopoietic stem/progenitor cell self-renewal (TGF-β; Fig. 1 H). Key functional genes (lymphotoxin β and ID3) are highly expressed by most fetal skin T cells (Fig. 1 H and data not shown) and may be important for providing differentiation signals within developing skin to epithelial cells, endothelial cells, and fibroblasts as well as regulation of T cell lineages. TGF-β and IFN-γ are expressed at much lower levels, and their expression appears restricted to individual cells among the SP αβ, SP γδ, and DP αβγδ T cells. However, no convincing T cell subclustering can be obtained based on TGF-β and IFN-γ expression. Furthermore, the expression of TGF-β and IFN-γ appears mutually exclusive, suggesting some degree of functional specialization of SP αβ, SP γδ, and DP αβγδ T cells (data not shown). In line with this, some genes are exclusively expressed in SP (IL-7, CCR8, and TGF-α) and DP T cells (IL-18 and TWIST1; Fig. 1 I).

**Figure 2.**
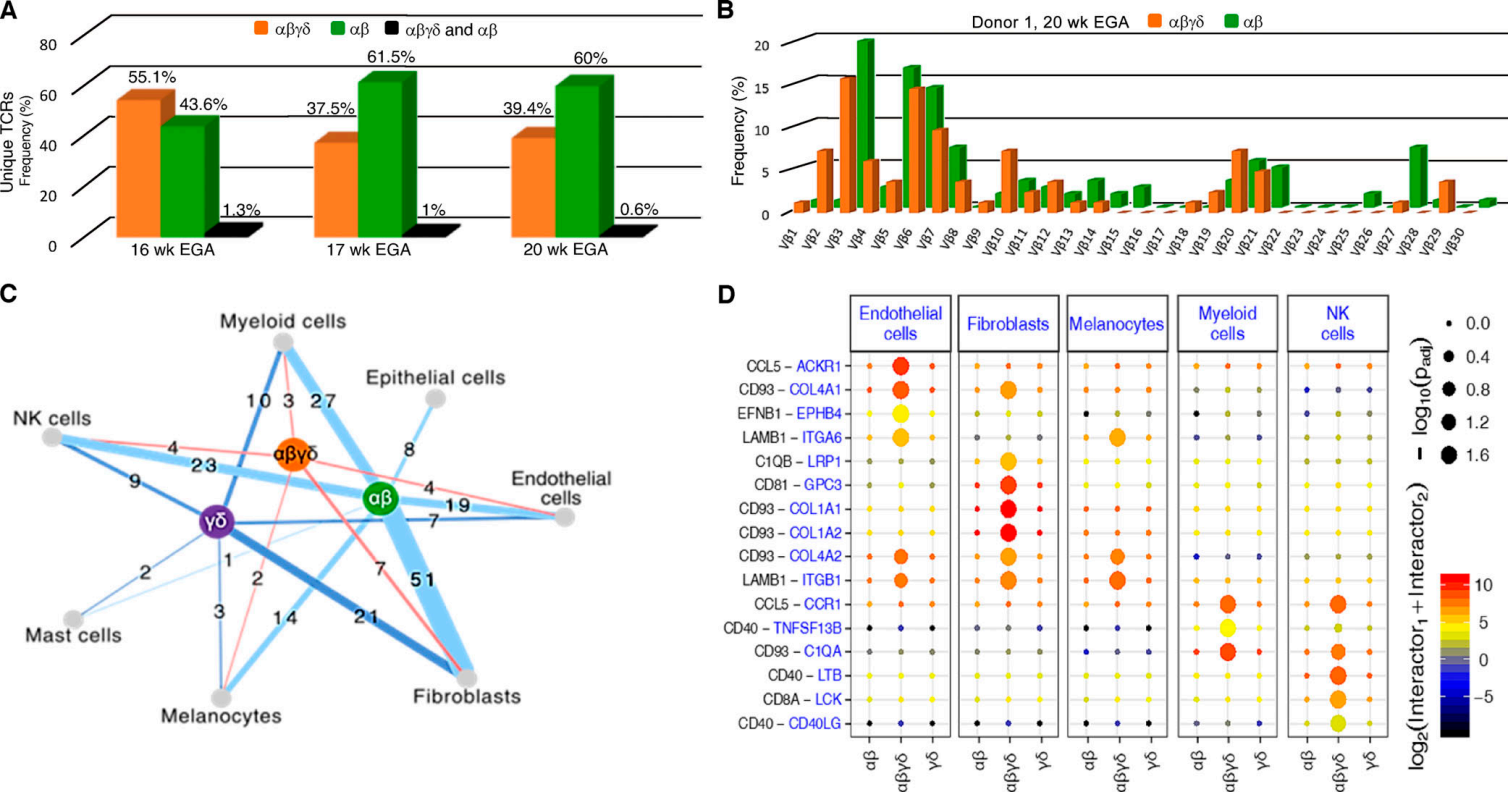
**TCR sequencing data and in silico receptor–ligand interaction studies. (A)** Comparative analysis of CDR3 sequences demonstrate rearrangements exclusively in DP αβγδ T cells (orange), SP αβ T cells (green), and both fetal skin T cell subsets (black; *n* = 3). **(B)** Frequency distribution of DP αβγδ and SP αβ T cell clones according to their constituent Vβ family member in one of three donors. **(C)** Cell networks with potential interactions of T cell subsets in fetal skin (*n* = 3). **(D)** Overview of selected TCR–ligand interactions; P values indicated by circle size; scale on right (permutation test). Means of the average expression level of interactions are indicated by color. Only droplet data were used (*n* = 3 biological replicates).

**Figure S1.**
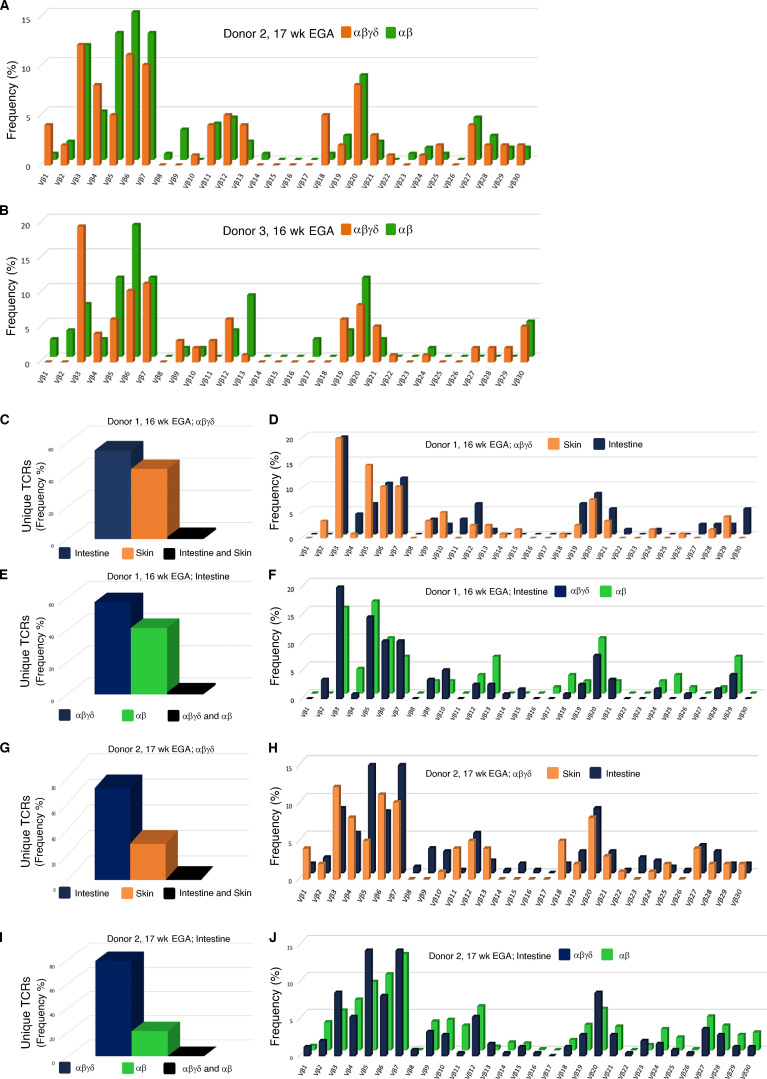
**High-throughput TCR sequencing analysis of flow-sorted T cell subsets in fetal skin and intestine. (A and B)** Frequency distribution of DP αβγδ and SP αβ T cell clones according to their constituent Vβ family member (*n* = 2).** (C–J) **Comparative analysis of the CDR3 frequency (C, E, G, and I) and TCR Vβ repertoire (D, F, H, and J) of flow-sorted DP αβγδ and SP αβ T cells in fetal skin and intestine (*n* = 2).

High-throughput TCR Vβ sequencing of the CDR3 region was performed with flow-sorted DP αβγδ and SP αβ fetal skin T cells. The majority of the rearranged TCR clones did not overlap (Fig. 2 A, orange and green bars), and only small numbers of unique TCRs were present in both DP αβγδ and SP αβ cells (Fig. 2 A, black bars). In addition, the distribution of Vβ families in both DP αβγδ and SP αβ indicates the presence of disparate T cell types in paired skin samples and across different fetuses (Fig. 2 B; and Fig. S1, A and B). It remains to be determined how this clonal diversity translates into distinct antigen recognition during cutaneous immunosurveillance.

To understand how T cell subsets interact with other (immune) cells in fetal skin, we next inferred cell-to-cell interactions based on the expression of annotated receptor–ligand pairs (Vento-Tormo et al., 2018). Among T cell subsets, SP αβ T cells showed stronger interactions with almost all skin cell populations compared with DP αβγδ and SP γδ T cells (Fig. 2 C). In contrast, inferred interactions of DP αβγδ T cells suggested an active role during fetal skin immune homeostasis/immune responses via interactions with other immune cells (myeloid cells, natural killer cells; Fig. 2 D). In particular, high expression of CD93 and the contact with ligands (Col1A1, Col1A2, Col4A1, and Col4A2) on fibroblasts and endothelial cells suggest that DP αβγδ T cells participate in skin embryogenesis (Fig. 2 D).

**Figure 3.**
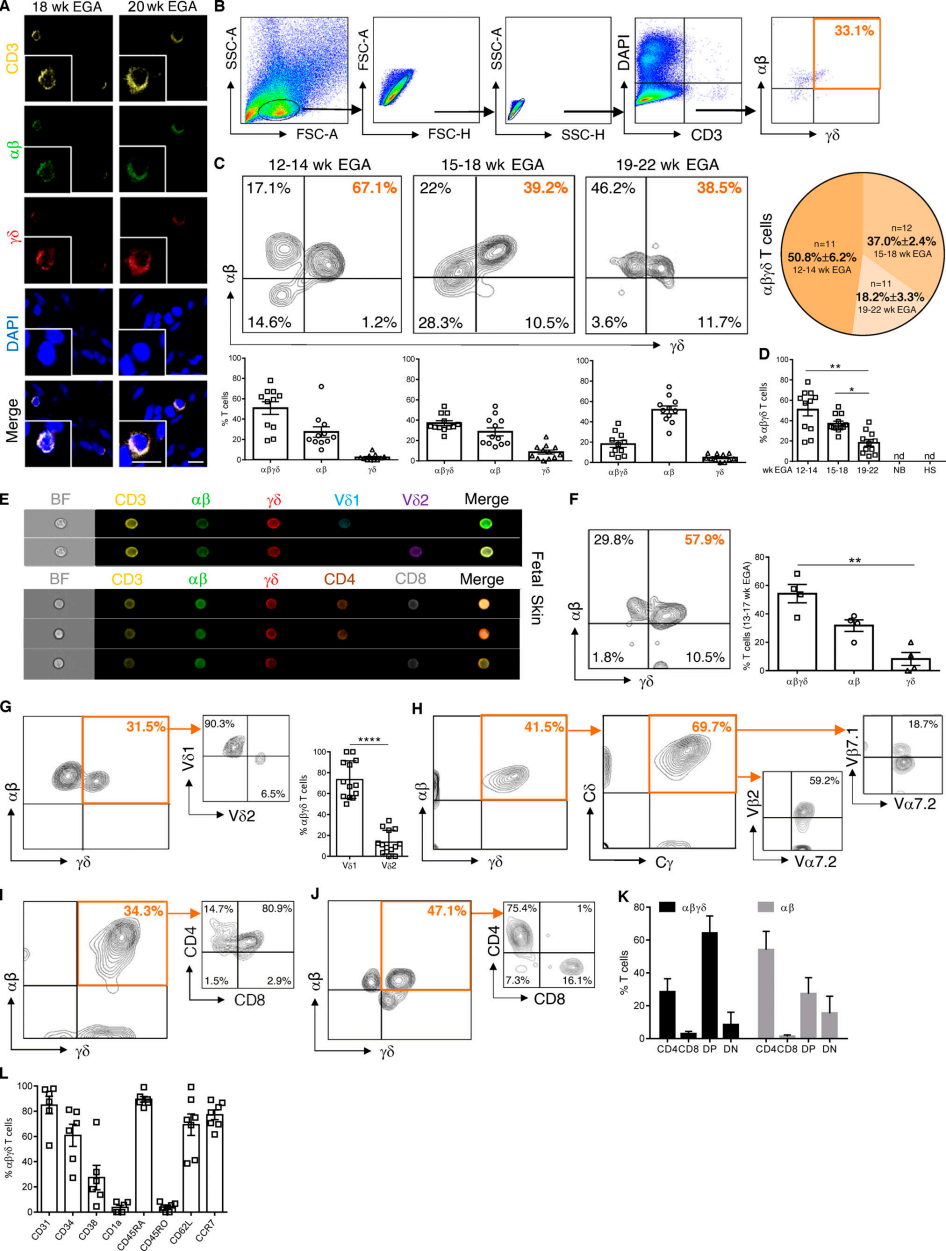
**Fetal skin harbors an exclusive T cell subset. (A)** Representative confocal microscopy images of whole-mount fetal skin showing DP αβγδ T cells in situ (*n* = 9). Scale bars, 10 µm. **(B)** Biaxial plots demonstrating the gating strategy for DP αβγδ T cells. **(C)** Representative plots showing DP αβγδ fetal skin T cells analyzed by flow cytometry. Kinetics and quantification of fetal skin T cell subsets during gestation (*n* = 34). **(D)** Bars revealing DP αβγδ T cells during gestation compared with newborn skin (NB, *n* = 3) and hypospadiasis skin (HS; *n* = 3). Each data point in the scatter plots represents an individual experiment and donor. **(E and F)** Imaging flow cytometry of DP αβγδ fetal skin T cells for indicated markers (*n* = 4, 13–17 wk EGA) and quantification of fetal skin T cell subsets. **(G)** Representative contour plots and percentage of Vδ1 and Vδ2 expression on DP αβγδ T cells in fetal human skin (*n* = 13). **(H)** Representative plots showing DP αβγδ T cells analyzed for the expression of δ/γ constant (identifying TCR γδ) and selected Vα/Vβ families (identifying TCR αβ; *n* = 5). **(I–K)** Percentage of CD4^−^CD8^−^ (DN), CD4^+^CD8^+^ (DP), CD4^+^, and CD8^+^ T cells within DP αβγδ and SP αβ T cells (*n* = 25). **(L)** Characterization of DP αβγδ T cells for indicated markers. Tukey's multiple comparison test; *, P < 0.05; **, P< 0.01; ****, P < 0.0001. Mean ± SEM.

**Figure S2.**
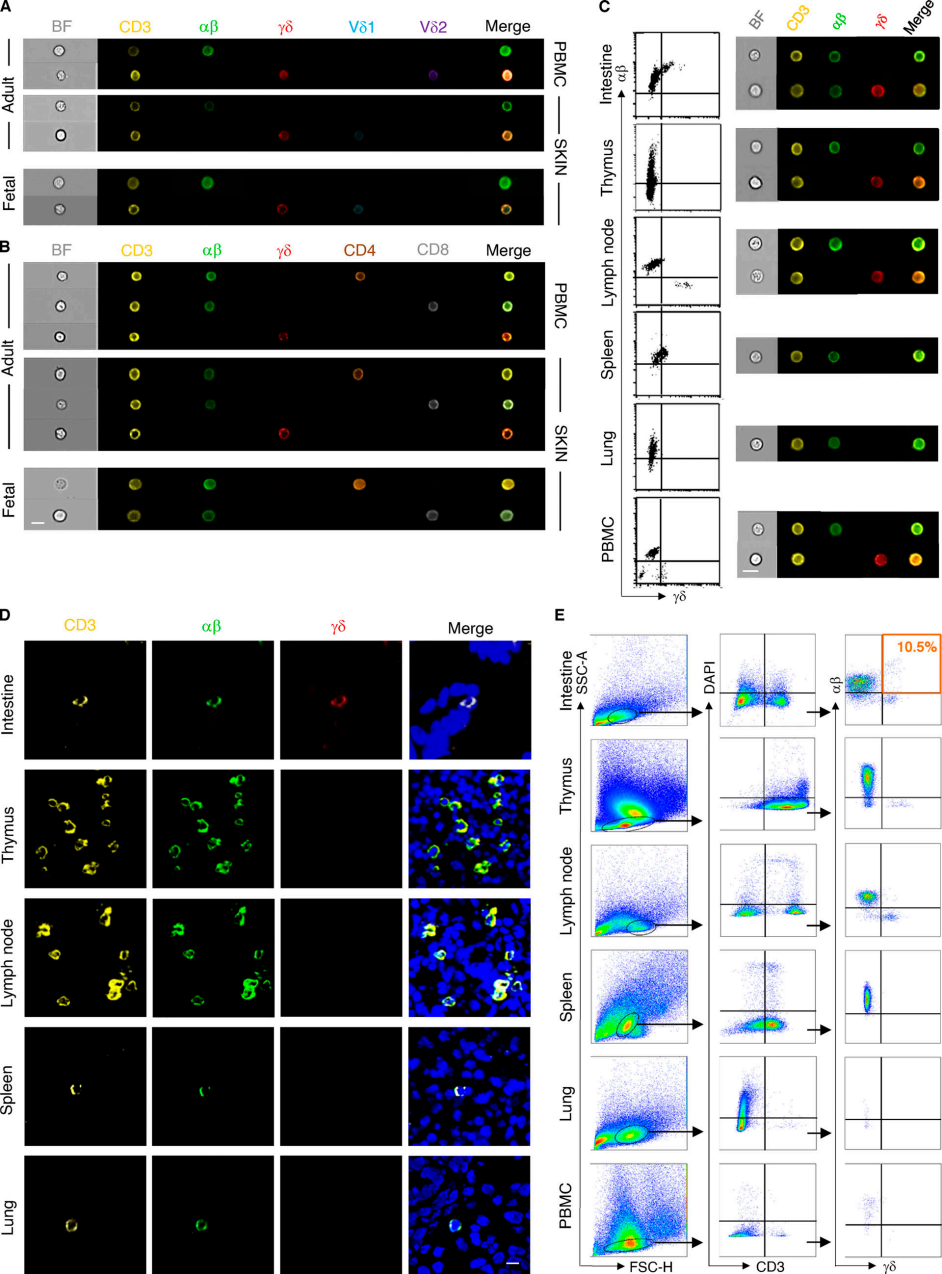
**Comparative analysis of T cells in fetal organs and PBMCs. (A and B)** Representative images of cell types showing brightfield (BF) and immunofluorescence (CD3/αβ/γδ/Vδ1/Vδ2/CD4/CD8) as analyzed using ImageStream (*n* = 4). **(C and E)** Representative biaxial plots demonstrating the gating of T cell subsets in fetal organs and PBMCs as well as analysis by ImageStream and flow cytometry (*n* = 5). **(D)** Representative confocal laser microscopy images showing a T cell marker expression profile in indicated fetal organs (*n* = 4). Scale bar, 10 µm.

To further validate our transcriptional findings during organ development over time, we explored the presence of DP αβγδ T cells at different developmental stages using microscopy and flow cytometry. Application of triple immunofluorescence staining on skin sections detected DP αβγδ T cells in second trimester fetal skin exclusively in the dermis in situ (Fig. 3 A). Time course experiments of TCR surface expression showed that the frequency of DP αβγδ T cells (of total CD3^+^ T cells) decreased significantly during gestation from 51% (12–14 wk) to 18% (19–22 wk; Fig. 3, B and C). DP αβγδ T cells were absent in newborn skin at term delivery and noninflamed foreskin samples 1–2 yr after birth (Fig. 3 D). Percentages of cutaneous SP αβ T cells increased during gestation, whereas SP γδ T cells remained present at low frequency (Fig. 3 C; Schuster et al., 2012). This is comparable to T cell frequencies in healthy adult human skin, with αβ T cells making up the remaining population (Cruz et al., 2018). Using ImageStream, the presence of DP αβγδ T cells was corroborated in fetal but never in adult skin cell suspensions or fetal and adult peripheral blood mononuclear cells (PBMCs; Fig. 3, E and F; and Fig. S2, A–C). Analysis of other fetal organs uncovered DP αβγδ T cells in intestine but not in thymus (Park et al., 2020), mesenteric lymph nodes, spleen, and lung (Fig. S2, C–E). Instead, we observed an abundance of SP αβ T cells and low frequency or absence of SP γδ T cells in all investigated organs (Fig. S2, C–E). Comparative analysis of TCR sequencing data of flow-sorted DP αβγδ and SP αβ T cells revealed a divergent presence of clones in skin and intestine of paired samples and across fetuses (Fig. 2, A and B; and Fig. S1, A–J).

**Figure S3.**
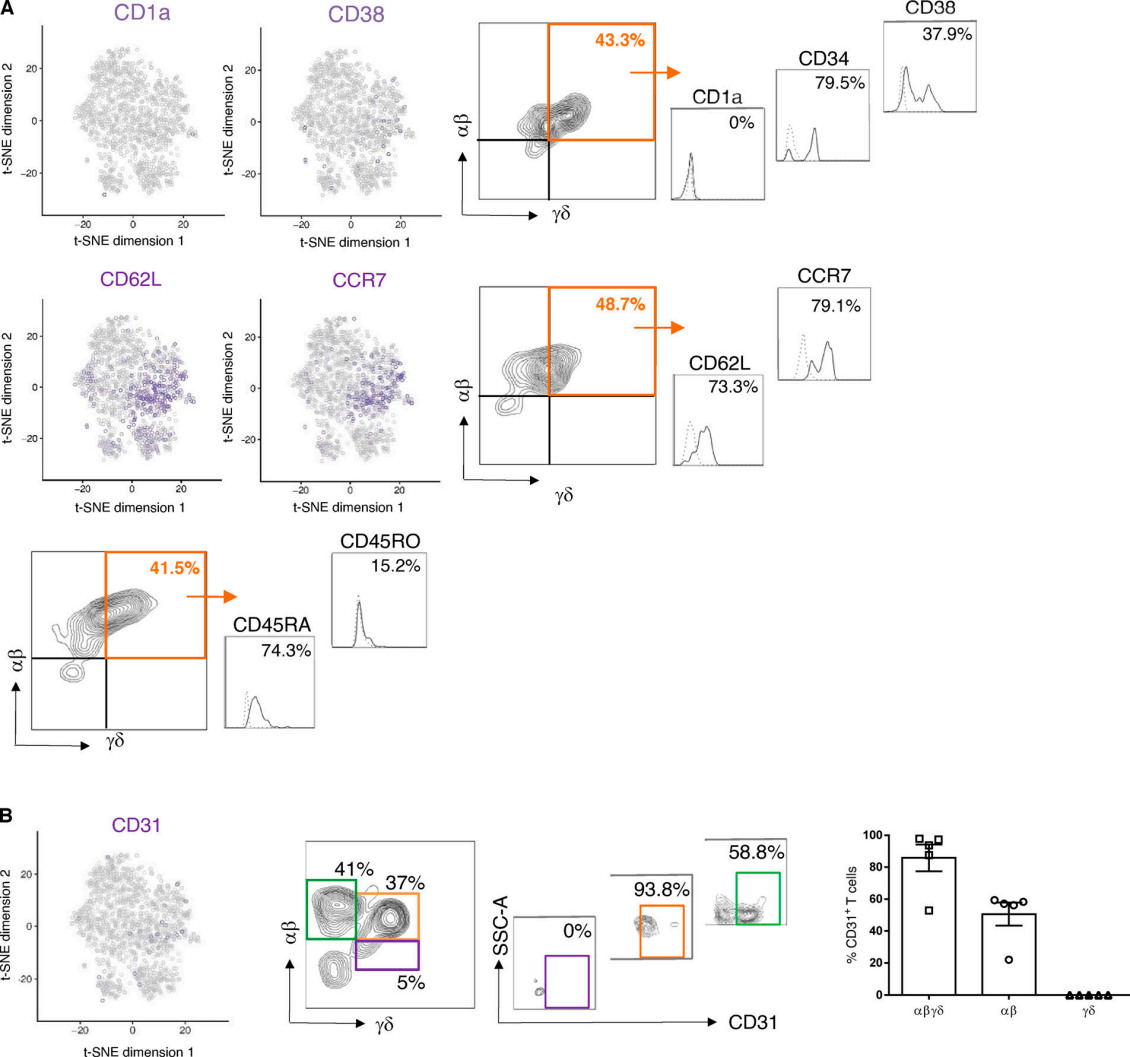
**Phenotypic characterization of DP αβγδ T cells in fetal human skin.** Biaxial plots and histograms depict one representative experiment. **(A and B)** t-SNE blots of indicated genes in DP αβγδ fetal skin T cells. Histograms show expression of selected hematopoietic stem cell and progenitor markers on DP αβγδ fetal skin T cells analyzed by flow cytometry (*n* = 5–7).

SP γδ T cells in adult individuals are divided into tissue-associated Vδ1 T cells that can pair with a variety of Vγ chains and peripheral blood–associated Vδ2 T cells (Cruz et al., 2018; Davey et al., 2018). We found that the majority (76%) of DP αβγδ fetal skin T cells expressed the Vδ1 chain (Fig. 3, E and G). Vδ2 T cells, representing the predominant γδ T cell subset in human fetal blood and thymus at 20 wk EGA (Papadopoulou et al., 2019; Dimova et al., 2015), were significantly less frequent compared with Vδ1 among DP αβγδ fetal skin T cells (Fig. 3, E and G). DP αβγδ fetal skin T cells expressed fully rearranged αβ and γδ TCRs, as the staining of the γδ constant regions and selected αβ family members including Vβ2, Vβ7.1, and Vα7.2 showed a high frequency of cells positive for both receptors (Fig. 3 H). The majority of DP αβγδ T cells and a minority of SP αβ T cells coexpressed CD4 and CD8 in early second trimester fetal skin (12–14 wk; Fig. 3, E, I, and K; and Fig. S2 B). A trend toward SP CD4^+^ and CD8^+^ DP αβγδ T cell populations was observed during later development (Fig. 3, J and K). DP αβγδ skin T cells expressed surface markers that are characteristic for naive T cells and recent thymic emigrants (CD45RA and CD31), hematopoietic stem cells (CD34 and CD38), and T cell progenitors (CD62L and CCR7; Fig. 3 L and Fig. S3, A and B). Even though CD31 expression is a hallmark for the identification of recent thymic emigrants (Douaisi et al., 2017), our finding that DP αβγδ T cells were undetectable in the thymus provides evidence for extrathymic development of DP αβγδ T cells in skin and intestine (Fig. 3 L; Fig. S2, C–E; and Fig. S3, A and B).

**Figure 4.**
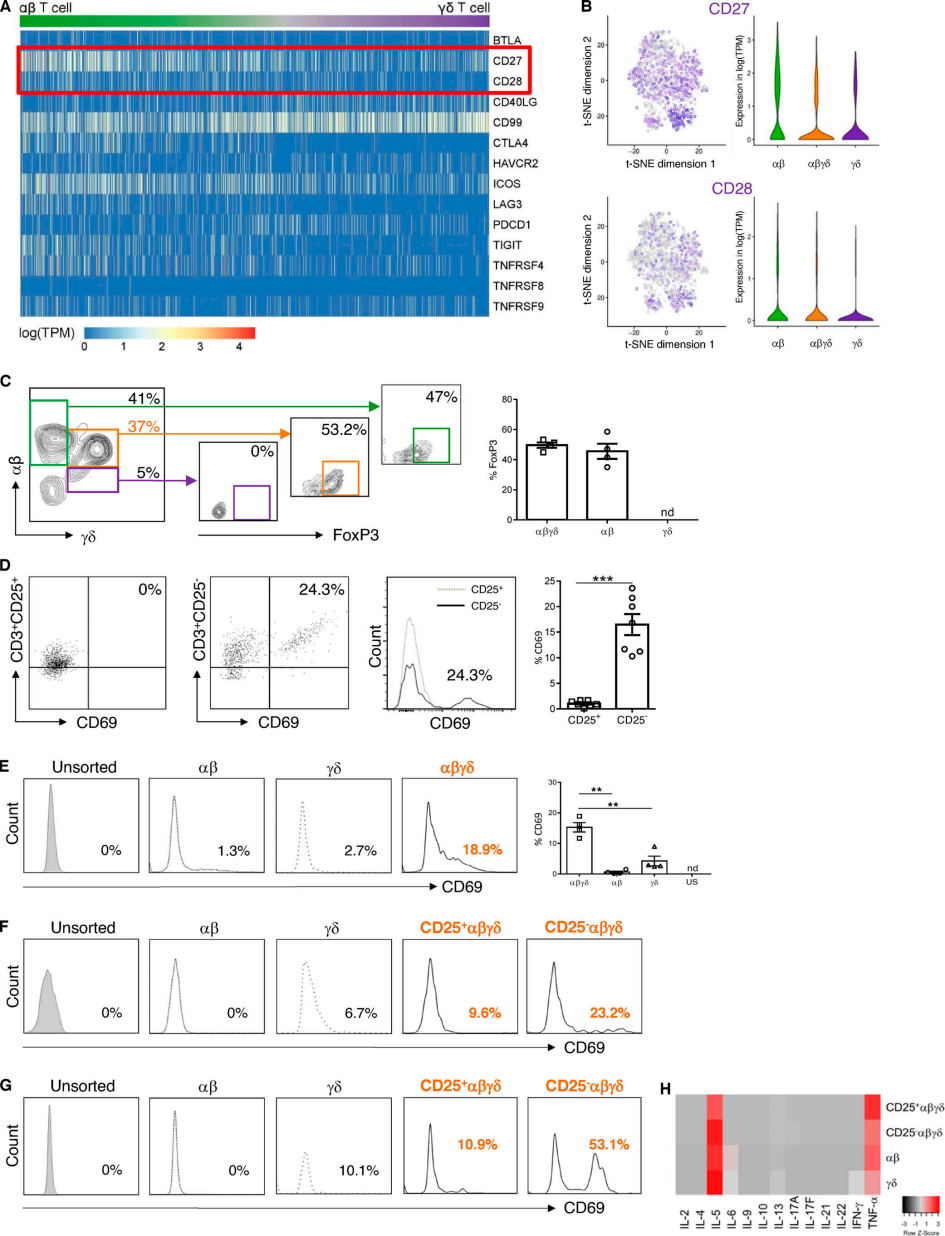
**Functional profiling of fetal skin T cell subsets. (A)** Heatmap showing normalized single-cell gene expression values for key costimulatory and coinhibitory molecules. **(B)** t-SNE blots presenting CD27 and CD28 (log-normalized RNA, violin plots) in fetal skin T cell subsets (*n* = 4). **(C)** Representative plots showing FoxP3 expression in T cell subsets analyzed by flow cytometry (*n* = 4). nd, not detectable. **(D–G)** Total fetal skin T cells and flow-sorted T cell subsets, depleted of CD25^+^ T cells or not, were stimulated with anti-CD3/CD27 mAbs (D–F) or PMA/ionomycin (G). Expression of CD69 was determined by flow cytometry (*n* = 3). **(H)** Cytokine concentrations in supernatants of PMA/ionomycin-stimulated cultures were determined by cytokine bead array in duplicates. US, unsorted; nd, not detectable. Tukey's multiple comparison test: **, P < 0.01; ***, P < 0.001. Mean ± SEM.

To assess functional properties of human fetal skin T cells, they were analyzed for their ability to respond to TCR ligation, based on antibody-coated beads and plate-bound and soluble antibodies directed against key costimulatory molecules (Chen and Flies, 2013) with high/intermediate (CD27) and low (CD28) gene expression (Fig. 4, A [red box] and B). Of note, gene expression of other costimulatory molecules was identified in DP αβγδ T cells (CD40L and CD99), while coinhibitory genes (CTLA-4 and PD1) were hardly ever expressed (data not shown). Irrespective of the stimulation protocol and antibodies used, no activation of fetal skin T cells was observed. We noticed the presence of CD25^+^Foxp3^+^ T reg cells within SP αβ and DP αβγδ T cells in fetal skin (Fig. 4 C). Given that T reg cells prevent T cell stimulation (Byrne et al., 1994; Michaëlsson et al., 2006), CD25^+^T cells were depleted from total fetal skin cell suspensions. Using anti-CD3/CD27 stimulation, T cells were activated in the absence but not in the presence of T reg cells, as evidenced by upregulation of the early activation antigen CD69 on T cells (Fig. 4 D). Exposure of flow-sorted T cell subsets to the same stimulus showed that significantly more DP αβγδ T cells were activated compared with SP αβ and SP γδ T cells (Fig. 4 E). Moreover, upon depletion of CD25^+^αβγδ T cells, a markedly enhanced activation of CD25^−^αβγδ T cells was observed upon stimulation (Fig. 4, F and G). These data show that CD25^+^ αβγδ T cells actively suppress the activation of CD25^−^αβγδ T cells in vitro and may also promote immune suppression in utero. All T cell subsets produced a large array of cytokines (regulatory, T helper–related as well as proinflammatory cytokines [IL-2/4/5/6/9/10/13/17A/17F/21/22, IFN-γ, and TNF-α]) upon PMA/ionomycin stimulation. Of note, common to all T cell subsets was a higher secretion of TNF-α and IL-5 compared with other cytokines (Fig. 4 H). Importantly, recent studies have highlighted the contribution of TNF-α in tissue development and immunity, as TNF-α–producing T cells promote intestinal epithelial tissue growth in early human fetal life (Schreurs et al., 2019), and human fetal dendritic cells foster immune suppression and impair T cell TNF-α production in response to allogeneic antigens through arginase-2 (McGovern et al., 2017). A role for IL-5 in immunoregulatory processes has been implied based on its fundamental involvement in T reg cell function (Tran et al., 2012).

**Figure 5.**
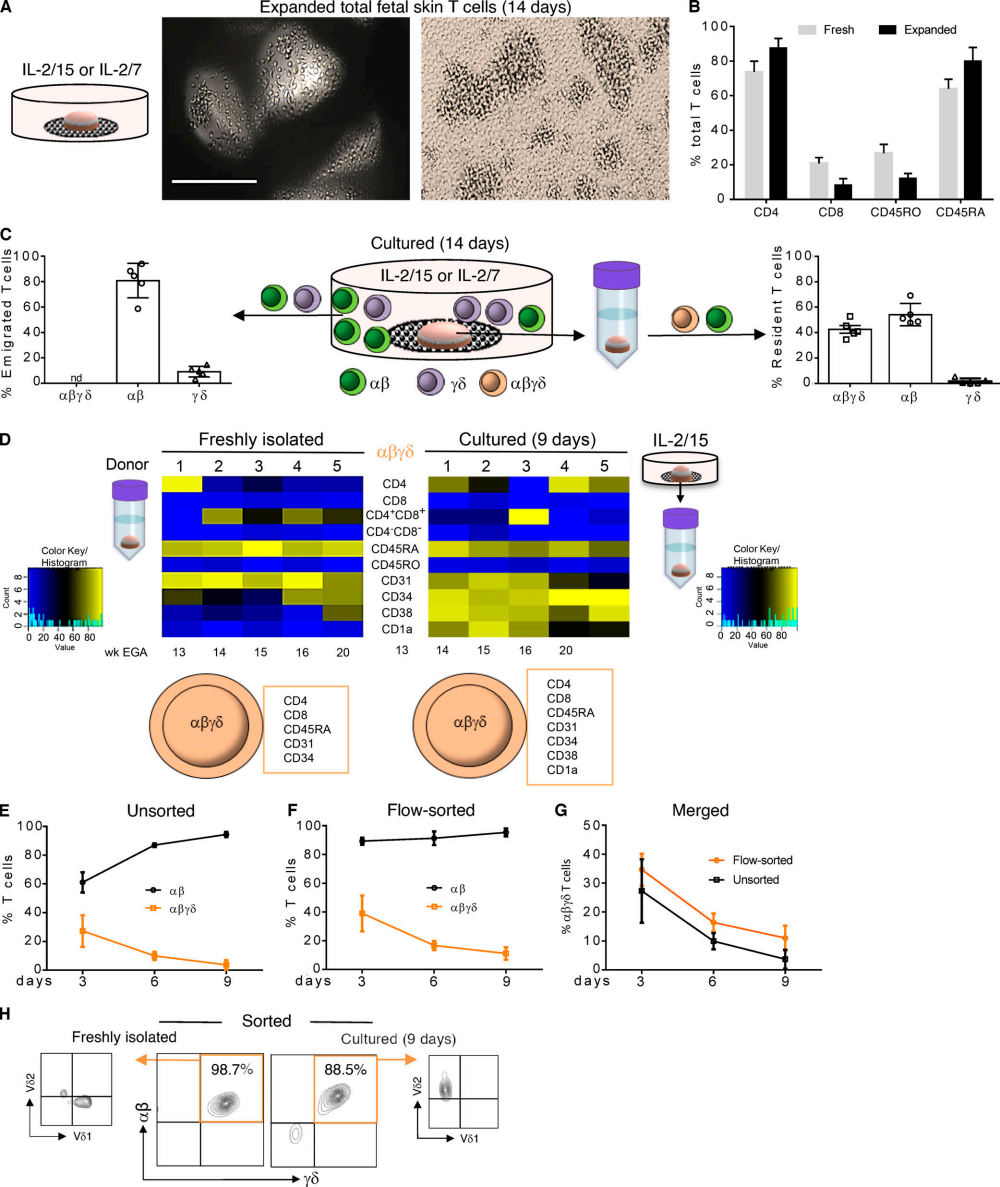
**Migration and expansion potential of fetal skin T cells ex vivo. (A)** Upon culture of fetal skin biopsies on grids, huge T cell clusters were observed after 14 d. Scale bar, 200 µm. **(B)** Comparative analysis of freshly isolated and expanded fetal skin T cells using flow cytometry and indicated markers (*n* = 5). **(C)** Frequency of T cell subsets emigrated and expanded from fetal skin biopsies (left) and isolated from skin biopsies (right) after 14 d of culture. Mean ± SEM; nd, not detectable (*n* = 5). **(D)** Heatmap showing expression of indicated markers on DP αβγδ T cells isolated before culture and upon 9 d of organ culture (*n* = 5). **(E–G)** DP αβγδ in contrast to SP αβ fetal skin T cells did not expand in IL-2/15 conditioned medium. Unsorted, 13–21 wk EGA, *n* = 7; flow-sorted, 13–20 wk EGA, *n* = 5. **(H)** Vδ2 but not Vδ1 DP αβγδ fetal skin T cells can be expanded in the presence of IL-2 and zoledronate (*n* = 5).

To test the expansion potential of fetal T cells, skin biopsies were placed on the surface of collagen-coated, 3D cell growth matrices and cultured in serum-free medium with cytokines of the common γ-chain family (Clark et al., 2006). Irrespective of the cytokine combination, several T cell clusters were observed after 2 wk (Fig. 5 A). Flow cytometry analysis revealed that expanded T cells preserved the expression of markers such as CD4, CD8, and CD45RA, as their expression levels were similar to freshly isolated fetal skin T cells (Fig. 5 B). Remarkably, SP αβ and γδ T cells were present consistently in culture wells, while DP αβγδ T cells were absent (Fig. 5 C). Analysis of skin cell suspensions from 2-wk cultured skin biopsies uncovered DP αβγδ T cells, demonstrating their inability to emigrate out of the skin (Fig. 5 C). A subsequent phenotypic comparison of DP αβγδ T cells in single-cell suspensions prepared from skin biopsies before and after organ culture revealed consistent expression of TCRs, CD4, CD8, and CD45RA, while other markers initially not expressed or only weakly expressed were upregulated during cultivation (CD34, CD38, and CD1a; Fig. 5 D). These data suggest that the skin environment drives DP αβγδ T cells toward a more differentiated phenotype, even though we cannot exclude that culture conditions, removal of amniotic fluid, and/or a lack of access to the fetal circulation (including several factors in fetal blood) might play a role as well. In addition, we asked whether DP αβγδ and SP αβ T cells can be expanded in fetal skin single-cell suspensions with IL-2/15. Comparable to organ cultures, we found vigorous expansion of SP αβ T cells. In contrast, DP αβγδ T cells virtually disappeared within 9 d (Fig. 5 E). Similar results were obtained with flow-sorted T cell subsets, showing that IL-2/15 promotes only the expansion of SP αβ T cells but not DP αβγδ T cells (Fig. 5, F and G). Given that DP αβγδ fetal skin T cells express either Vδ1 or Vδ2 (Fig. 3, E and G), a TCR γδ T cell expansion protocol with IL-2 and zoledronate, the most potent member of the bisphosphonate family of drugs, was applied (Kondo et al., 2011). Treatment of flow-sorted DP αβγδ T cells induced exclusive expansion of DP αβγδ Vδ2-type T cells upon 9 d of culture (Fig. 5 H). As DP αβγδ T cells were not identified in newborn skin (Fig. 3 D), and thus do not represent a population faced with postnatal microbes, their reactivity to phosphoantigens could reflect high concentrations of endogenous phosphoantigens (e.g., isopentyl pyrophosphate) derived from the fetal isoprenoid metabolism (Kondo et al., 2011). However, in case of invasions with (E)-4-hydroxy-3-methyl-but-2-enyl pyrophosphate–producing pathogens (Eberl et al., 2003; Remington et al., 2006), DP αβγδ T cells may provide immune defense via interaction with dendritic cells and promote conventional T cell responses (Ismaili et al., 2002; Fiore et al., 2007).

### General discussion and conclusions

Our study uncovered the presence of a nonmigratory T cell subset in human fetal skin with an exclusive TCR repertoire consisting of αβγδ chains that displayed no or negligible overlap of CDR3 sequences with SP αβ T cells. We show that these cells are not inactive but exist in a dynamic balance between activation and quiescence. Further, our data indicate that this balance is regulated by the presence of CD25^+^αβγδ T cells, as removal of this population resulted in a substantial activation of CD25^−^αβγδ T cells in response to stimulation. In line with our finding are recently published data describing a T cell population coexpressing the αβ and γδ TCR in mice that act as “first responders” during bacterial infection (Edwards et al., 2020). However, it is currently unclear how stimulation during intrauterine infections can override immune suppression mechanisms. Our data also suggest that DP αβγδ T cells could contribute to skin formation during development based on in silico receptor–ligand interaction studies, cytokine profile, and expression of particular granzymes (data not shown), known to degrade extracellular matrix proteins (Anthony et al., 2010). These observations support the concept (Schreurs et al., 2019) that T cells play a crucial role in the highly controlled process of fetal tissue development.

Recent investigations have provided evidence that the microenvironment of the developing human skin supports differentiation of immune cells, analogous to hematopoietic tissues (Botting and Haniffa, 2020). As for T cells, this has not been shown yet, and further studies will determine whether and how programming of human DP αβγδ T cells occurs in the skin and intestine. A growing body of evidence suggests that T cells may develop at extrathymic sites in mice (Arcangeli et al., 2005; Guy-Grand et al., 2003) and in humans (Dejbakhsh-Jones et al., 1995; García-Ojeda et al., 2005; Maillard et al., 2006; McClory et al., 2012), while many aspects (e.g., precursors, site, routes) of extrathymic T cell development are still elusive. Together, our findings provide new insights into the complexities of the developing adaptive skin immune system under physiological conditions. Future studies are aimed at dissecting the role of these peculiar cells in the course of intrauterine infections and inborn errors of immunity, as well as their potential for therapeutic interventions during pregnancy.

## Materials and methods

### Human samples and consent

The study on fetal tissues for research was approved by the local ethics committee of the Medical University of Vienna and conducted in accordance with the Declaration of Helsinki Principles. Women gave written informed consent for the donation of fetal tissue. All fetal (12–22 wk EGA) organs for this study (skin, lung, thymus, spleen, intestine, and lymph node) and blood were obtained after legal termination of pregnancy. For comparison across fetal organs, the same donors were always used. Adult skin (25–51 yr) was obtained from healthy volunteers after abdominal and thigh cosmetic surgery with approval from the local ethics committee of the Medical University of Vienna.

Buffy coats from peripheral blood of healthy adult volunteers were purchased from the local transfusion service (24–55 yr; Red Cross). PBMCs were isolated as interface cells after density gradient centrifugation, and erythrocytes were removed with ammonium chloride (0.8% NH_4_Cl/0.1 mM EDTA). PBMCs from human fetal umbilical blood were isolated by gradient density centrifugation (2,000 rpm, 25 min) by Ficoll (Ficoll-Plaque Plus, d = 1.077 g/ml; GE Healthcare) and washed two times with DPBS (1,500 rpm, 10 min).

### Cell isolation

For the preparation of single-cell suspensions of fetal organs, a dissociation kit in combination with an automatic tissue dissociator (gentleMACS Octo Dissociator; Miltenyi Biotec) was used according to the manufacturer’s instruction. Briefly, the dissociation kit samples (three biopsies, 4 mm) were put onto gentleMACS for mechanical treatment. The “h_skin_01” preinstalled program was used. Cell clumps and tissue debris were removed with a 70-µm nylon cell strainer, and remaining cells were further processed for indicated procedures.

### Single-cell RNA-sequencing (scRNA-seq)

Single-cell libraries were generated using the Chromium Controller and Single Cell 3′ Library & Gel Bead Kit v2 (10x Genomics) according to the manufacturer’s protocol. Briefly, viable CD3^+^ T cells (up to 5,000 cells/fetus) were FACS sorted from single-cell suspensions of three fetal (17–22 wk EGA) skin donors. To get a more complete resolution of human skin development, we additionally sort-purified up to 10,000 total skin cells and mixed them with the CD3^+^ T cell population. Cells were pelleted by centrifuging for 5 min at 4°C, 300 *g* and resuspended in PBS with 0.04% BSA. Up to 10,000 cells suspended in reverse transcription reagents, along with gel beads, were segregated into aqueous nanoliter-scale gel beads in emulsions (GEMs). The GEMs were then reverse transcribed in a C1000 Thermal Cycler (Bio-Rad) programmed at 53°C for 45 min, 85°C for 5 min, and hold at 4°C. After reverse transcription, single-cell droplets were broken, and the single-strand cDNA was isolated and cleaned with Cleanup Mix containing Dynabeads MyOne SILANE (Thermo Fisher Scientific). cDNA was then amplified with a C1000 Thermal Cycler programmed at 98°C for 3 min; 10 cycles of 98°C for 15 s, 67°C for 20 s, 72°C for 1 min; 72°C for 1 min; and hold at 4°C. Subsequently, the amplified cDNA was fragmented, end-repaired, A-tailed, and index adapter ligated, with cleanup in between steps using SPRIselect Reagent Kit (Beckman Coulter). Postligation product was amplified with a T1000 Thermal Cycler programmed at 98°C for 45 s; 10 cycles of 98°C for 20 s, 54°C for 30 s, 72°C for 20 s; 72°C for 1 min; and hold at 4°C. The sequencing-ready library was cleaned up with SPRIselect beads and sequenced by the Biomedical Sequencing Facility at the Center for Molecular Science using the Illumina HiSeq 3000/4000 platform and the 75-bp paired-end configuration.

### scRNA-seq data preprocessing

Preprocessing of the scRNA-seq data was performed using Cell Ranger (Zheng et al., 2017; v2.1.0) from 10x Genomics. Raw sequencing files were demultiplexed using the Cell Ranger command mkfastq. Each sample was aligned to the human reference genome assembly refdata-cellranger-GRCh38-1.2.0 using the Cell Ranger command count, and all samples were aggregated using the Cell Ranger command aggr without depth normalization. Cell types were assigned based on visual inspection of marker gene expression in the loupe browser provided by 10x Genomics. Raw expression data of T cells were loaded into R (v3.4.0) and further analyzed using the Seurat package (v2.3.0). Raw data were normalized to transcripts per million (TPM), log transformed, and scaled with Seurat using default parameters (Butler et al., 2018). To integrate data across donors, canonical correlation analysis was performed using Seurat. 10 canonical correlation analysis components were used to generate a t-SNE representation of T cells.

### T cell subset assignments

T cell subsets (αβ and γδ T cells) were initially assigned based on expression of the constant chain of the TCR (TRAC, TRBC1, TRBC2, TRDC, TRGC1, and TRGC2), and then refined using a machine learning approach. As β subunits were expressed across all T cells subsets, only subunits α, δ, and γ were used. Cells with high expression of subunit α (log TPM > 2.5 for TRAC) and low expression of all δ and γ subunits (log TPM < 0.5 for all of TRGC1, TRGC2, and TRDC) were labeled as αβ T cells. Cells with low expression of subunit α (log TPM of TRAC < 0.5) and high expression of any δ or γ subunit (log TPM > 2.5 for any of TRGC1, TRGC2, or TRDC) were labeled as γδ T cells. Cells with high expression of subunit α (log TPM of TRAC > 2.5) and high expression of any δ or γ subunit (log TPM > 2.5 for any of TRGC1, TRGC2, or TRDC) were labeled as DP T cells. All remaining cells were assigned to T cell subsets based on a machine learning predictor. To do so, a logistic regression model was trained to predict T cell subset (αβ or γδ T cell) from log TPM expression data of each cell for each donor separately. Models were trained on expression data of initially assigned αβ and γδ T cells, after expression data of all genes encoding constant subunits of the TCRs were removed. Data from donors 1 and 2 were used for training, because other donors did not provide enough γδ T cells. Logistic regression models were trained using elastic net regularization through the glmnet package in R. Observations were weighted to reflect imbalance in the data. A nested cross-validation was used to assess prediction accuracy. Data were split fivefold for the outer loop. Within each fold, a 10-fold inner validation loop was performed on 80% (fourfold) of data using the function cv.glmnet. The best model, as determined by the inner loop, was then tested on 20% (onefold) of data. In addition to nested cross-validation, prediction accuracy was evaluated on data from other donors, by training models on all data from one donor and applying them on other donors. Finally, trained predictors were applied to previously unassigned cells to obtain class probability scores, which denote whether a cell belongs to γδ T cells (probability = 1) or αβ T cells (probability = 0). Predictions of the two models from donors 1 and 2 were averaged. Cells with large scores (probability > 0.75) were assigned as γδ T cells, cells with low scores (probability < 0.25) were assigned to αβ T cells, and cells with intermediate probability (0.25 < probability < 0.75) were assigned as DP cells. Differential expression of αβ and γδ T cells was performed using the function FindConservedMarkers of the Seurat package with the negative binomial distribution test, a minimum log fold-change of 0.1, and otherwise default parameters. Genes with significant change (adjusted P < 0.05) in donors 1 and 2 were used as markers of the two T cell subsets.

### Cell–cell interaction analysis

Cell–cell interactions were inferred based on a methodology from Vento-Tormo et al. (2018). We thus tested for annotated receptor–ligand pairs, whether the two interaction partners are highly expressed in a particular combination of T cell and non–T cell. Annotated receptor–ligand pairs were obtained from a dataset published by Ramilowski et al. (2015) (filtered to literature-supported interactions) and the database CellPhoneDB (Vento-Tormo et al., 2018). We calculated a score for the combined expression data from both genes, with data from one interactor (e.g., receptor) taken from the T cell and data from the other interactor (e.g., ligand) taken from the non–T cell. Scores were calculated by averaging the TPM normalized expression of the two genes and log2 transforming. Only genes with reads in at least 20% of cells in any cell type were considered. Next, we assessed whether these scores were significant for the specific combination of cell types. To do so, we generated two background distributions, once controlling for expression in T cells and once controlling for expression in other cells. We thus calculated the same scores, once after exchanging the T cells in question by a randomly drawn set of other T cells, and once by exchanging the non–T cells by a randomly drawn set of other non–T cells. Random draws were performed 1,000 times, P values were calculated using the function t.test in R, combined across the two background sets by taking the maximum P value, and adjusted using the function p.adjust with method BH.

### DNA isolation and high-throughput sequencing

Total DNA was isolated from FACS-sorted T cell subsets (fetal skin and intestine) using the DNA Mini Kit 50 (51304; Qiagen). High-throughput TCR sequencing of the Vβ chain was performed using the ImmunoSEQ kit (Adaptive Biotechnologies) according to manufacturer’s instructions. CDR3 sequences of paired samples were compared to determine the frequency of common clones. TCR reads were plotted according to their Vβ family type.

### Immunofluorescence

Tissue specimens were embedded in an optimal cutting temperature formulation (Tissue Tek), snap frozen in liquid nitrogen, and stored at –80°C until further processing. 5-μm sections were cut, mounted on capillary gap microscope slides, fixed in ice-cold acetone for 10 min, and air dried. Subsequently, sections were incubated in a humid chamber with recombinant antibodies (1:50; 1 h, 4°C; Table S1). After washing with PBS (2× 5 min), slides were stained for 1 min with DAPI, washed with PBS (2× 5 min), mounted with Fluoprep (bioMérieux), and analyzed with a confocal laser scanning microscope (LSM 410; Carl Zeiss).

### Flow cytometry and cell sorting

Multicolor flow cytometry for surface and intracellular markers (Table S1) was performed on Aria II/III or FACS Verse (BD Biosciences), and data were analyzed using FlowJo software (TreeStar; V_10). The gating strategy included discrimination of doublets and dead cells. Appropriate isotype controls were included. T cell subsets were sorted (up to 99% purity) from freshly digested tissue cell suspensions by FACS using Aria II/III.

### Imaging flow cytometry

Cells were stained with defined combinations of recombinant antibodies (Table S1). Dead cells were excluded with DAPI and recorded with an ImageStreamX Mark II Imaging Flow Cytometer (Luminex Corp.). Data were analyzed with IDEAS (Image Data Exploration and Analysis Software; Merck Millipore) and FlowJo.

### T cell stimulation

Several stimulation protocols have been tested for fetal skin T cells (18–22 wk EGA). First, freshly isolated fetal skin cells were cultured in 96-well round-bottom plates (Merck) with TexMACS medium, IL-2 (2 ng/ml; both Miltenyi Biotec), and anti-CD3/CD28 mAb-coated microbeads (bead:cell ratio 1:1, Human T-Activator CD3/CD28, GIBCO BRL Dynabeads; Thermo Fisher Scientific). Second, freshly isolated fetal skin cells were cultured in 96-well flat-bottom plates (Beckton Dickinson Labware Europe) coated with an anti-CD3 mAb (10 µg/ml; BD Bioscience) and a soluble anti-CD28 mAb (3 µl/10^6^ cells; BD Bioscience) in TexMACS medium and IL-2 for 3 d. Third, unsorted and flow-sorted fetal skin T cell subsets were cultured in the presence of IL-2 in 96-well flat-bottom plates in TexMACS medium with and without plate-bound anti-CD3 mAb (10 µg/ml; BD Bioscience) and a soluble anti-CD27 mAb (3 µl/10^6^ cells; BD Bioscience) for 3 d. To bypass TCR/CD3 activation, unsorted fetal skin cells and flow-sorted T cell subsets were stimulated with PMA and ionomycin (1:500; Thermo Fisher Scientific) for 3 h. Irrespective of the stimulation protocol, cultured cells were analyzed for the expression of the activation marker CD69 by flow cytometry.

### T cell isolation from skin explant cultures

Cellfoam matrices (grids, 9 × 1.5 mm; Cytomatrix) were autoclaved and incubated in PBS (1× 10 ml) and collagen G (250 µl, 30 min, room temperature; Biochrom). Punched (4 mm; Kai Europe) skin samples were cut into small pieces (∼1 mm) and transferred onto the grids. The charged grids were transferred into wells of a 24-well plate (Becton Dickinson Labware Europe) containing 2 ml TexMACS medium (1% penicillin/streptomycin) without or with a combination of cytokines (100 U/ml IL-2; PeproTech; 5 ng/ml IL-15/IL-7; Miltenyi Biotec). After 9–14 d of cultivation, cells were harvested and centrifuged (7 min, 4°C) and the supernatant aspirated and frozen. Cell pellets were resuspended in 500 µl PBS and the resulting single-cell suspensions analyzed by flow cytometry. Isolation of cells from cultured skin biopsies was performed as described.

### Cell culture with bisphosphonate zoledronate

Total fetal skin cell suspensions and flow-sorted T cell subsets (1–10 × 10^5^) were seeded into 96-well round-bottom plates in 200 µl TexMACS medium and IL-2 (100 U/ml; PeproTech), with or without bisphosphonate zoledronate (5 µM; Fresenius Kabi). On indicated days (Fig. 5 H), cells were harvested for counting and analysis by flow cytometry.

### Analysis of cytokine concentrations

Cytokine production of flow-sorted T cells upon stimulation with PMA/ionomycin was assessed (Fig. 4 H) using bead array analysis with LegendPlexHumanTh Cytokine Panel (13-plex; BioLegend). The assay was performed according to the manufacturer’s instructions. Cytokine concentrations were calculated using LegendPlex v8.0 data analysis software (BioLegend).

### Statistical analyses

Statistical analysis used for each experiment is described in the figure legends. Each *n* number represents an individual donor and a separate experiment. The software used for statistical analyses was GraphPad Prism 6.01, and P values of <0.05 were considered significant. No statistical methods were used to predetermine sample size. The experiments were not randomized.

### Data availability

Sequence data that support the findings of this study have been deposited in GEO under accession no. GSE156972.

### Online supplemental material

Fig. S1 shows high-throughput TCR sequencing analysis of flow-sorted T cell subsets in fetal skin and intestine. Fig. S2 shows comparative analysis of T cells in fetal organs and PBMCs. Fig. S3 shows a phenotypic characterization of DP αβγδ T cells in fetal human skin. Table S1 shows all antibodies used in this study.

## Supplementary Material

Table S1 lists all antibodies used in this study.
