# Supplementary material for: αβγδ T cells play a vital role in fetal human skin development and immunity
Source: J Exp Med. 2021 Feb 9;218(4):e20201189. doi: 10.1084/jem.20201189 (PMC7876551; doi:10.1084/jem.20201189)
Supplement: Table S1 — lists all antibodies used in this study. [file JEM_20201189_TableS1.docx]

Table S1 **Antibodies**

| **Antigen** | **Clone** | **Conjugation** | **Amount^a^** | **Isotype** | **Supplier** |
| --- | --- | --- | --- | --- | --- |
| CD3 | REA613  REA613 | APC  PerCP-Vio700 | 1 μl | Recombinant human IgG1 | Miltenyi Biotech |
| CD4 | REA623 | VioGreen | 1 μl | Recombinant human IgG1 | Miltenyi Biotech |
| CD8 | REA734 | APC-H7 | 1 μl | Recombinant human IgG1 | Miltenyi Biotech |
| αβ TCR | REA652 | FITC | 1 μl | Recombinant human IgG1 | Miltenyi Biotech |
| γδ TCR | REA591 | PE-Cy7  PE | 1 μl | Recombinant human IgG1 | Miltenyi Biotech |
| Vδ1 | REA173  REA173 | APC PE | 1 μl | Recombinant human IgG1 | Miltenyi Biotech |
| Vδ2 | REA771 | PE | 1 μl | Recombinant human IgG1 | Miltenyi Biotech |
| CD45RA | HI100 | PE | 1.2 μl | Mouse monoclonal IgG2a, κ | Becton Dickinson |
| CD45RO | UCHL1 | PerCP | 1.3 μl | Mouse monoclonal IgG2a | Thermofischer |
| CD34 | REA1164 | PerCP-Vio700 | 1 μl | Recombinant human IgG1 | Miltenyi Biotech |
| CD38 | REA671 | PE-Vio770 | 1 μl | Recombinant human IgG1 | Miltenyi Biotech |
| CD1a | REA736 | APC-Vio770 | 1 μl | Recombinant human IgG1 | Miltenyi Biotech |
| CD62L | DREG-56 | PE-Cyanine7 | 1 μl | Mouse monoclonal IgG1,κ | Thermo Fisher Scientific |
| CCR7 | REA546 | PerCp-Vio700 | 1 μl | Recombinant human IgG1 | Miltenyi Biotech |
| CD31 | REA730 | APC | 1 μl | Recombinant human IgG1 | Miltenyi Biotech |
| CD25 | REA570 | APC | 1.5 μl | Recombinant human IgG1 | Miltenyi Biotech |
| CD69 | FN50 | Pe-Cy7 | 1 μl | Mouse monoclonal IgG1, κ | Thermo Fisher Scientific |
| FoxP3 | 259/C7 | PE | 3 μl | Mouse monoclonal IgG1, κ | Becton Dickson |

^a^ amount per 10^5^/100 μl
